# methyLImp2: faster missing value estimation for DNA methylation data

**DOI:** 10.1093/bioinformatics/btae001

**Published:** 2024-01-11

**Authors:** Anna Plaksienko, Pietro Di Lena, Christine Nardini, Claudia Angelini

**Affiliations:** Oslo Centre for Biostatistics and Epidemiology, Department of Biostatistics, University of Oslo, Oslo 0317, Norway; Department of Computer Science and Engineering, University of Bologna, Bologna 40126, Italy; Institute for Applied Mathematics (IAC) “Mauro Picone”, National Research Council (CNR) Rome, Rome 00185, Italy; Institute for Applied Mathematics (IAC) “Mauro Picone”, National Research Council (CNR) Naples, Naples 80131, Italy

## Abstract

**Motivation:**

*methyLImp*, a method we recently introduced for the missing value estimation of DNA methylation data, has demonstrated competitive performance in data imputation compared to the existing, general-purpose, approaches. However, imputation running time was considerably long and unfeasible in case of large datasets with numerous missing values.

**Results:**

*methyLImp2* made possible computations that were previously unfeasible. We achieved this by introducing two important modifications that have significantly reduced the original running time without sacrificing prediction performance. First, we implemented a chromosome-wise parallel version of *methyLImp*. This parallelization reduced the runtime by several 10-fold in our experiments. Then, to handle large datasets, we also introduced a mini-batch approach that uses only a subset of the samples for the imputation. Thus, it further reduces the running time from days to hours or even minutes in large datasets.

**Availability and implementation:**

The R package methyLImp2 is under review for Bioconductor. It is currently freely available on Github https://github.com/annaplaksienko/methyLImp2.

## 1 Introduction

*methyLImp* was introduced in Di Lena *et al.* (2019, 2020) as a method for missing value imputation specific for DNA methylation array data. It relies on solving numerous multiple linear regressions that enhance the high inter-sample correlation observed at the methylation levels. In comparative studies, *methyLImp* demonstrated good performances and computational efficiency, but improvements were required to reduce the running time in the case of large datasets. Therefore, with *methyLImp2*, we introduce two major implementation changes that, together with other improvements, decrease the running time significantly. The first one is the parallelization of the algorithm over chromosomes. Correlation among CpGs methylation within a chromosome, owing to the DNA and chromatin spatial distribution, is a well-known pattern of methylated data (Zhang *et al.* 2009). Therefore, we decided to impute missing values on CpGs using only probes on the same chromosome. We split the data by chromosomes and run the algorithm independently in parallel for each sub-dataset. The second improvement consists of an optional mini-batch approach to cut down the computation time further, now sample-wise [inspired by Hicks *et al.* (2021), whose approach is based on Sculley (2010)]. Taken together, these improvements allow using *methyLImp2* also with large EPIC datasets.

*methyLImp2* is implemented as an R package methyLImp2, which is tested on multiple platforms, including Windows, Mac, and Linux, and is freely available on GitHub (under review for Bioconductor, unlike the previous version).

## 2 Materials and methods

### 2.1 methyLImp

In this section, we briefly describe the original *methyLImp* algorithm [Di Lena *et al.* (2019)], and we highlight the modifications we have introduced in the further sections.

Methylation levels exhibit high inter-sample correlations that multiple linear regression models can exploit. Therefore, *methyLImp* estimates missing values by performing simultaneous linear regressions with pseudo-inverse on the corresponding sets of available data. Assume we have an n×m input matrix of methylation levels (β-values) with *n* observations/samples (in rows) of *m* CpG sites/probes (in columns). First, we identify all *L* variables (i.e. CpG sites) that contain missing values. Then, we group the variables with the same pattern of missingness, i.e. those with missing values for the same observations (samples). We call that set of *r* rows $R_{NA}$ and that set of *l* columns $C_{NA}$. *methyLImp* aims to estimate the missing values simultaneously for each group of variables, resulting in an r×l submatrix of imputed values *Imp*. To obtain *Imp*, a linear regression is applied simultaneously to the whole group of columns $C_{NA}$ by solving the following:

$$A\cdot x=\text{logit}(B)\Rightarrow x=A^{-1}\cdot \text{logit}(B)\Rightarrow \mathrm{Imp}={\text{logit}}^{-1}(C\cdot x),$$

where

-
A is the (n−r)×(m−L) submatrix of all non-missing variables for all the observations not in $R_{NA}$;
-
C is the r×(m−L) submatrix of all non-missing variables for all the observations in $R_{NA}$;
-
B is the (n−r)×l submatrix of all variables in $C_{NA}$ for all the observations not in $R_{NA}$;
-
logit(p)=log(p/(1−p)) , p∈[0,1], and its inverse logistic function ${\text{logit}}^{-1}(q)=1/(1-\text{exp}(-q)))$, q∈(−∞,∞).

The above procedure is applied to each group of columns with the same NA pattern. Therefore, the algorithm’s computational costs depend on the number of groups with the same pattern of missing values (i.e. the number of regression problems to solve) and the dataset’s dimensions *n* and *m*. Those dimensions get quite large when dealing with genome-wide methylation data. For example, the HumanMethylation450 BeadChip evaluates over 450 000 probes for each sample, and the Infinium Methylation EPIC v2.0 BeadChip has over 850 000 sites. Hence, solving all the linear problems can be computationally intense. Therefore, we thought of two ways to reduce the dimensionality of the linear systems, both column-wise—splitting CpG sites and parallelizing the calculations over chromosomes—and row-wise—implementing a mini-batch approach that uses a reduced number of samples.

### 2.2 Parallelization over chromosomes

As mentioned above, methylation data is characterized by the large number of variables (probes). We assumed that information from all 450 000 or, even more, 850 000 probes may be unnecessary for precise imputation on one probe. Moreover, it seems plausible that probes on the same chromosome may provide more valuable input. Therefore, we split the CpG sites over chromosomes and then apply the *methyLImp* algorithm to each chromosome separately. This modification significantly reduces the number of columns *m* in all the matrices of each regression problem. Although we will show in the next section that reducing matrices’ dimensions is already enough to improve the runtime, we parallelize computations for each sub-dataset and benefit from multiple cores. We implemented parallelization in methyLImp2 with BiocParallel package [Morgan *et al.* (2023), version 1.36.0] and achieved a greater improvement in the running time. Since chromosomes differ widely in size, we have used load balancing for parallel computations. With this approach, the distribution of tasks to workers (cores) is not pre-determined from the start but is dynamic, meaning that the task is assigned to the core that becomes available first.

### 2.3 Mini-batch

To further improve the running time of *methyLImp2*, we implemented the mini-batch strategy to be used when the datasets consist of a large number of samples *n*. To reduce the dimensions of the matrices A and B, the mini-batch approach uses only a fraction *P* of the available samples for each linear regression. This decreases runtime for all matrix manipulations, especially for heavy operations such as multiplication. The pseudocode of the algorithm is presented below.

Algorithm 1. Mini-batch algorithm pseudocode

INPUT: data X, sample size *n*,

percentage of samples chosen P←10/20/30,

number of repetitions R←1/2/3.

**for**r=1 to *R* **do**

 $X_{P}\leftarrow$ Random Sample of X of the Size n/P*100;

 $Imp_{r}\leftarrow$*methyLImp*($X_{P}$);

**end for**

Average the results over *R*, $\mathrm{Imp}\leftarrow \sum_{r}Imp_{r}/R$.

OUTPUT: Imputed elements Imp.

Note that Algorithm 1 focuses only on the mini-batch procedure. In the actual code, X is first split into matrices A,B,C, as described above, and only A and B are subsampled. That is done independently before each linear regression (i.e. for each group of columns, CpG sites, with the same missingness pattern). If the number of rows in A is already smaller than n/P*100 by construction, we do not perform the subsampling.

### 2.4 Other improvements

We have also introduced a few additional improvements to the code. For the identification of columns with the same missingness pattern, we used unique and identical base R functions. For faster SVD decomposition, we used fast.svd from corpcor package [Schafer *et al.* (2021), version 1.6.10], which is specifically faster for matrices with a large difference in magnitude of the number of samples *n* and the number of variables *m*. To help streamline the analysis process for Bioconductor users, methyLImp2 now also accepts the SummarizedExperiment class as an input.

### 2.5 Practical considerations

The user should consider the following points to benefit from methyLImp2 fully. According to intuition and best practice, when a macroscopic characterization of the samples is available as subgroups, e.g. therapeutic groups, experimental or environmental conditions, we suggest applying *methyLImp2* to each group independently. In methyLImp2, users can specify the sample groups when multiple conditions are available. The dataset is then split into sub-datasets with (more) homogeneous conditions, and *methyLImp2* (i.e. chromosome-wise probes splitting, column groups identification, imputation) is applied to each sub-dataset independently.

The mini-batch procedure is advised for *large* datasets: e.g. in the following sections, we demonstrate its advantage with a dataset with 450+ samples. In general, we suggest considering mini-batch when the number of samples is on the order of hundreds, although it can be used for smaller sizes with caution. However, in such cases, retaining a large proportion of samples is advisable.

We refer the reader to Supplementary data for some additional considerations.

### 2.6 Datasets

We utilized two large datasets to assess the prediction performance and running time of *methyLImp2*.

The GSE199057 Gene Expression Omnibus (GEO) dataset contains 68 mucosa samples from non-colon-cancer patients. Methylation data intensities were measured on EPIC arrays and transformed as β-values. We used minfi (Aryee *et al.* 2014) package to remove SNPs loci. We also removed unmapped probes and probes mapping to sex chromosomes. Resulting dataset contains 816 126 probes. There are 2843 NA entries in the dataset, i.e. 0.005%.

The GSE158063 GEO dataset contains EPIC array methylation measurements of the peripheral blood buffy coat of individuals at the 26–28 gestational weeks of pregnancy. To reduce heterogeneity in the dataset, we have limited our focus to women of Chinese ethnicity, 25 to 40 years of age, who had spontaneous conception and gave live birth. This dataset contains β-values for 456 samples and 816 117 probes (probes filtering performed as described above). There are 11 654 NA entries in the dataset, i.e. 0.003%.

Both datasets had less than 1% of probes containing at least one missing value. To assess the performance of the method, we amplified that and randomly chose 3% of *m* probes to have artificial NAs. Then, for each probe, we randomly defined the number of NAs from a Poisson distribution with λ appropriate to the sample size of the dataset (see examples in the next section). Finally, these NAs were randomly placed among the *n* samples. With this procedure, most columns had a unique missing values pattern and did not group, thus providing a worst-case running time due to the high number of regression problems to solve. If we had more groups, i.e. fewer individual linear regressions, the running time reported below would be lower. Ultimately, the missing values account for approximately ≈0.4% of entries of the 68 samples dataset and ≈0.2% of entries of the 456 samples dataset. Note that this paper does not discuss the maximum amount of NAs allowed to perform the imputation. We refer the reader to the previous *methyLImp* manuscript for performance studies with 10, 30, 50, and even 70 % missing values Di Lena *et al.* (2019). Although performance unsurprisingly deteriorates with percentage increase, we note that with *methyLImp* it happens at a much slower rate than with its competitors. Above all, we encourage users to consider whether a large number of NAs in a sample may indicate low quality and whether discarding that sample would be wiser.

We store the positions of the artificial NAs to be able to distinguish them from the “natural” ones for the performance evaluation later. We refer the reader to methyLImp2 package for implementation of the NA generation procedure and the simulation studies code for more details. We repeated the NA generation procedure 5 times for the 68 samples dataset and 3 times for the 456 samples dataset to get independent datasets for testing—from here on, running time and accuracy performance are reported on averages.

To access the performance, we evaluate root mean square error and mean absolute error, defined as follows:

$$\mathrm{RMSE}=\sqrt{\frac{\sum_{\beta \in NA}{\left(\beta -\beta^{\mathrm{imp}}\right)}^{2}}{\left|NA\right|}},\mathrm{MAE}=\frac{\sum_{\beta \in NA}|\beta -\beta^{\mathrm{imp}}|}{\left|NA\right|},$$

where β and $\beta^{\mathrm{imp}}$ denote the true and the imputed β-values. Note that in the *NA* set only the artificial NAs are taken into account, as we cannot access the performance for the true ones since their values are unknown. For additional metrics, such as Pearson correlation coefficient (PCC) and mean absolute percentage error (MAPE), we refer the reader to the Supplementary data.

Simulations were carried out on an Apple M1 Max 10-core processor and 64 GB RAM computer. All simulation code and the datasets are available on Github github.com/annaplaksienko/methyLImp2_simulation_studies.

### 2.7 Results

**Figure 1.**
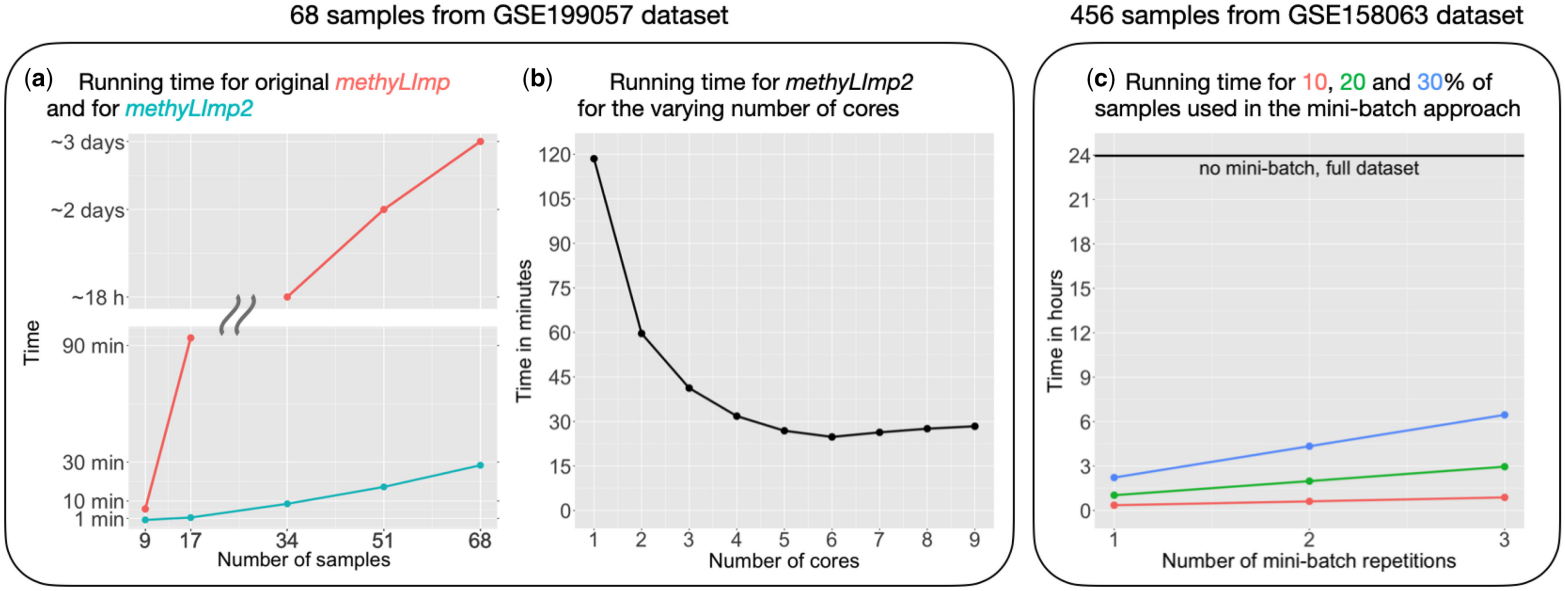
Running times for *methyLImp* and *methyLImp2* measured under various settings: (**a**) comparison of the running times for original *methyLImp* and for *methyLImp2* for different number of samples, (**b**) running time for *methyLImp2* for the varying number of cores and (**c**) running time for *methyLImp2* with different mini-batch settings.

We first compared the performance of original *methyLImp* with those of *methyLImp2* for various sample sizes *n*. We used a subset of the GSE199057 dataset consisting of 68 samples. We applied *methyLImp2* to randomly chosen 9, 17, 34, 51 samples and to all 68 samples with λ for Poisson distribution in NA generation procedure being set to 1, 2.5, 5, 7.5, and 10, respectively. Results are reported in Panel (a) of Fig. 1. As we can see, the difference in the running time is dramatic: for the entire dataset with 68 samples *methyLImp2* requires less than 30 minutes, while *methyLImp* needs approximately three days. Meanwhile, the difference in performance is negligible, and *methyLImp2* even performs better for 9 samples, see Table 1. Although we cannot definitively account for such performance gap, we can speculate that, since the number of features is higher in *methyLImp* than in *methyLImp2* (which splits the probes across chromosomes), while the number of samples is limited in both cases, background noise affects *methyLImp* regressions more than *methyLImp2*. As the number of samples increases, overall prediction performances tend to improve and converge. A more comprehensive table, which includes standard deviations, is provided in the Supplementary data.

**Table 1.** Root mean square error (RMSE) and mean absolute error (MAE) for original unparallelized version of *methyLImp* and parallelized over chromosomes *methyLImp2*.

No. of samples	methyLImp		methyLImp2	
	RMSE	MAE	RMSE	MAE
9	0.051	0.032	0.049	0.031
17	0.045	0.027	0.042	0.026
34	0.045	0.026	0.044	0.025
51	0.044	0.025	0.042	0.024
68	0.042	0.024	0.042	0.024

We emphasize that the running time gain in *methyLImp2* can be achieved using a small number of cores and even a single core since the splitting of probes (columns) over chromosomes allows to hold matrix operations in smaller dimensions. In Panel (b) of Fig. 1, we demonstrate the running time of *methyLImp2* applied to GSE199057 dataset with 68 samples, changing the number of cores from 1 to 9. As we can see, the runtime drops drastically (from 2 h to around 30 minutes) when increasing the number of cores. We also emphasize that significant improvement can be obtained even with only 4 cores, which, nowadays, even simple laptops have.

Interestingly, running time does not improve further with the number of cores above 5. One reason for that may be that overall running time is often determined by the computations on the core(s) with the largest chromosome(s), so after some point increasing the number of cores will not affect the time. That is not always the case, though, since the chromosome running time also depends on the amount and distribution of NAs. Therefore, the optimal task allocation and the choice of the number of cores is not straightforward. The current version of methyLImp2 package by default uses all physical cores except one. However, the user can change that number if needed.

To assess the advantages of the mini-batch approach, we applied *methyLImp2* to the subset of GSE158063 dataset with 456 samples with and without mini-batch and compared the running time and accuracy performance. As we can see from Panel (c) of Fig. 1, for the fastest option—using 10% of the data in the mini-batch and repeating the estimate only once—*methyLImp2* running time for imputing the entire dataset is only twenty minutes while without the mini-batch it takes a whole day (24 h) to process the entire dataset. Even when using 30% of data in the mini-batch and repeating the estimate 3 times, *methyLImp2* runs for about 7 h, so still less than for the entire dataset. Meanwhile, the accuracy performance (see Table 2) does not significantly decrease when using the mini-batch. On the contrary, in some cases, the accuracy of *methyLImp2* with the mini-batch is even better than what observed without the mini-batch for the entire dataset.

**Table 2.** Root mean square error and mean absolute error for different settings of mini-batch approach of *methyLImp2* and no mini-batch for comparison (last line with 100%).

% of samples	No. of mini-batch repetitions					
	1		2		3	
	RMSE	MAE	RMSE	MAE	RMSE	MAE
10%	0.0285	0.0172	0.0275	0.0168	0.027	0.0166
20%	0.0277	0.0167	0.0267	0.0163	0.0265	0.0162
30%	0.0274	0.0165	0.0265	0.0162	0.0263	0.0161
100%	0.0277	0.0166				

## 3 Discussion

In this work, we have enhanced *methyLImp* computational efficiency by several orders of magnitude, making it practical for imputing large datasets. We achieved this by implementing in *methyLImp2* the mini-batch approach and parallelization of computations over chromosomes.

Although performances in terms of running time are now acceptable to excellent even with large datasets, future improvements may include the following aspects. First, since chromosomes differ in sizes and some are very large, it could be helpful to add further parallelizations over different groups on the same chromosome, i.e. columns with the same missingness patterns. It would also be interesting to see what results we could achieve with random splitting of probes, not by chromosomes. Finally, parts of the code in C programming language could further improve the speed of matrix operations.

## Supplementary Material

btae001_Supplementary_Data

## Data Availability

Both datasets can be found on The Gene Expression Omnibus (GEO) dataset under accession numbers GSE199057 and GSE158063. Pre-processed datasets are available here and here. Simulation studies code is available on GitHub.
