# Supplementary material for: methyLImp2: faster missing value estimation for DNA methylation data
Source: Bioinformatics. 2024 Jan 11;40(1):btae001. doi: 10.1093/bioinformatics/btae001 (PMC10826905; doi:10.1093/bioinformatics/btae001)
Supplement: btae001_Supplementary_Data [file btae001_supplementary_data.pdf]

methyLImp2: faster missing value estimation for DNA methylation data
Supplementary Materials

Anna Plaksienko, Pietro Di Lena, Christine Nardini, Claudia Angelini

In this supplementary material, we provide additional metrics and running time for *methyLImp* method, both original and upgraded, as well as some practical considerations for the use of *methyLImp2* method and `methyLImp2` package.

1 Additional metrics

Let NA be a set of artificially introduced missing values. For each $\beta \in NA$ (meaning true value that we replaced by an artificial NA for the simulation purposes), β^{imp} denotes the corresponding imputed β-value. We define the following metrics for comparing true and imputed β-values:

- Root Mean Square Error (RMSE)

$$\text{RMSE} = \sqrt{\frac{\sum\limits_{\beta \in NA} (\beta - \beta^{imp})^2}{|NA|}};$$

- Mean Absolute Error (MAE)

$$\text{MAE} = \frac{\sum\limits_{\beta \in NA} |\beta - \beta^{imp}|}{|NA|};$$

- Pearson Correlation Coefficient (PCC)

$$\text{PCC} = \frac{\sum\limits_{\beta \in NA} (\beta - \overline{\beta})(\beta^{imp} - \overline{\beta^{imp}})}{\sqrt{\sum\limits_{\beta \in NA} (\beta - \overline{\beta})^2}\sqrt{\sum\limits_{\beta \in NA} (\beta^{imp} - \overline{\beta^{imp}})^2}}$$

  where $\overline{\beta}$ and $\overline{\beta^{imp}}$ are the sample mean of β and β^{imp}, respectively;

- Mean Absolute Percentage Error (MAPE)

$$\text{MAPE} = \frac{100}{n} \sum_{\beta \in NA} \left| \frac{\beta - \beta^{imp}}{\beta} \right|.$$

  When calculating MAPE, we omit the true β-values equal to 0, i.e. those for which $\beta = 0$, along with their corresponding predicted values. Including these values would result in an indeterminate measure.

Tables 1 and 2 contain the average and the standard deviation of described metrics over several runs for the comparison (Table 1) of *methyLImp* vs. *methyLImp2* for variying number of samples, and performance (Table 2) of *methyLImp2* with mini-batch approach (see the manuscript for simulations description).

	methyLImp				methyLImp2			
#Samples	RMSE	MAE	PCC	MAPE	RMSE	MAE	PCC	MAPE
9	0.051 ± 0.0005	0.032 ± 0.0002	0.986 ± 0.0003	19.29 ± 1.65	0.049 ± 0.0005	0.031 ± 0.0001	0.987 ± 0.0003	18.14 ± 1.30
17	0.045 ± 0.0013	0.027 ± 0.0003	0.989 ± 0.0007	16.83 ± 1.56	0.042 ± 0.0013	0.026 ± 0.0003	0.990 ± 0.0006	14.98 ± 0.61
34	0.045 ± 0.0005	0.026 ± 0.0001	0.988 ± 0.0002	19.32 ± 1.97	0.044 ± 0.0004	0.025 ± 0.0001	0.989 ± 0.0002	17.75 ± 1.47
51	0.044 ± 0.0005	0.025 ± 0.0002	0.989 ± 0.0003	17.77 ± 0.95	0.042 ± 0.0003	0.024 ± 0.0001	0.990 ± 0.0002	16.95 ± 0.62
68	0.042 ± 0.0001	0.024 ± 0.0001	0.990 ± 0.0001	16.56 ± 0.86	0.042 ± 0.0003	0.024 ± 0.0001	0.990 ± 0.0001	16.16 ± 0.82

Table 1: Average Root Mean Square Error (RMSE), Mean Absolute Error (MAE), Pearson Correlation Coefficient (PCC) and Mean Absolute Percentage Error (MAPE) $\pm$ standard deviation over 5 runs for the original unparallelized version of *methyLImp* and the parallelized version over chromosomes *methyLImp2* for various sample sizes. Note that the prediction accuracy improves for both approaches as the sample size increases, leading to convergence across all metrics.

Fraction of samples	#repetitions	RMSE	MAE	PCC	MAPE
10%	1	0.0285 ± 0.0001	0.0172 ± 0.00005	0.996 ± 0.00002	7.72 ± 0.05
10%	2	0.0274 ± 0.0001	0.0168 ± 0.00005	0.997 ± 0.00002	7.45 ± 0.06
10%	3	0.0270 ± 0.0002	0.0166 ± 0.00003	0.997 ± 0.00003	7.45 ± 0.07
20%	1	0.0276 ± 0.0002	0.0167 ± 0.00002	0.997 ± 0.00003	7.55 ± 0.08
20%	2	0.0269 ± 0.0002	0.0164 ± 0.00001	0.997 ± 0.00005	7.39 ± 0.09
20%	3	0.0265 ± 0.0002	0.0162 ± 0.00002	0.997 ± 0.00005	7.29 ± 0.05
30%	1	0.0274 ± 0.0002	0.0166 ± 0.00002	0.997 ± 0.00005	7.53 ± 0.03
30%	2	0.0265 ± 0.0002	0.0162 ± 0.00003	0.997 ± 0.00005	7.30 ± 0.05
30%	3	0.0263 ± 0.0001	0.0161 ± 0.00002	0.997 ± 0.00003	7.25 ± 0.05
100%	1	0.0277 ± 0.0002	0.0166 ± 0.00006	0.997 ± 0.00005	7.71 ± 0.03

Table 2: Average Root Mean Square Error (RMSE), Mean Absolute Error (MAE), Pearson Correlation Coefficient (PCC) and Mean Absolute Percentage Error (MAPE) $\pm$ standard deviation over 3 runs for various mini-batch setting for *methyLImp2*.

2 Running time

This section contains the running times in Tables 3, 4, 5 corresponding to Panels (a), (b) and (c) of Figure 1 in the manuscript. Simulations were carried on Apple M1 Max 10-core processor and 64 GB RAM computer.

Here we also note that we used `ggbreak` package [1] to be able to represent widely different running times of *methyLImp* and *methyLImp2* in one plot.

#samples	methyLImp	methyLImp2
9	6 min $\pm$ 5 sec	16 sec $\pm$ 1 sec
17	94 min $\pm$ 2 min	1 min $\pm$ 5 sec
34	18 h $\pm$ 23 min	9 min $\pm$ 14 sec
51	2 days $\pm$ 2 h	17 min $\pm$ 8 sec
68	3 days $\pm$ 5 h	28 min $\pm$ 16 sec

Table 3: Average running times $\pm$ standard deviations for *methyLImp* and *methyLImp2* for varying number of samples over 5 runs.

#cores	methyLImp2
1	2 h ± 1 min
2	1 h ± 20 sec
3	41 min ± 6 sec
4	32 min ± 9 sec
5	27 min ± 3 sec
6	25 min ± 17 sec
7	26 min ± 18 sec
8	28 min ± 15 sec
9	28 min ± 16 sec

Table 4: Average running times ± standard deviations for *methyLImp2* for varying number of cores over 5 runs.

	# mini-batch repetitions		
% of samples	1	2	3
10%	21 min ± 20 sec	37 min ± 13 sec	53 min ± 40 sec
20%	1 h ± 17 sec	2 h ± 35 sec	3 h ± 4 min
30%	2.2 h ± 1.5 min	4.3 h ± 1.5 min	6.5 h ± 3 min
100%	24 h ± 7 min		

Table 5: Average running times ± standard deviations of *methyLImp2* for different mini-batch settings and no mini-batch for comparison (last line with 100%) over 3 runs.

3 Practical considerations

In this section we provide some additional practical considerations for the use of *methyLImp2* method and `methyLImp2` package.

- In `methyLImp2` package the users have the option of providing their own annotation for probe splitting. This may be useful in various situations. In particular, this is the option that we currently suggest in the case of customized arrays.
- In the manuscript we do not address the potential issues that may arise from cross-reactive probes [2]. We conducted only a naive check of their influence: we obtained a list of cross-reactive probes from the `maxprobes` package and simply removed them from the 68 samples dataset with artifical NAs and re-run `methyLImp2`. We did not see an increase in performance of *methyLImp2*, hence we suppose they did not confound the signal too much, if at all. However, as we did not carry out extensive study, we advise users to consider custom annotation and data splitting if cross-reactive probes are an important concern of theirs.
- Performing imputation (and the whole analysis pipeline) in the case of batch effect is a challenging task. As `methyLImp2` allows the user to specify subgroups on which imputation will be performed independently, using that feature may be one option to avoid "mixing" of samples before batch correction.

References

[1] Shuangbin Xu et al. "Use ggbreak to Effectively Utilize Plotting Space to Deal With Large Datasets and Outliers". In: *Frontiers in Genetics* 12 (2021). DOI: `10.3389/fgene.2021.774846`.

[2] Wanding Zhou, Peter W. Laird, and Hui Shen. "Comprehensive characterization, annotation and innovative use of Infinium DNA methylation BeadChip probes". In: *Nucleic Acids Research* 45.4 (Oct. 2016), e22–e22. ISSN: 0305-1048. DOI: 10.1093/nar/gkw967.
